# Twenty Years of Dispersive Liquid–Liquid Microextraction: An Umbrella Review of Methodological Quality, Thematic Evolution, and Roadmap for Evidence Integration in Analytical Chemistry

**DOI:** 10.3390/molecules31111918

**Published:** 2026-06-02

**Authors:** Hakim Faraji, Adrián Conde Díaz, Álvaro Santana Mayor, Bárbara Socas-Rodríguez, Antonio V. Herrera Herrera

**Affiliations:** Departamento de Química, Área de Química Analítica, Facultad de Ciencias, Universidad de La Laguna (ULL), Avenida Astrofísico Francisco Sánchez s/n, 38206 San Cristóbal de La Laguna, Tenerife, Spain; acondedi@ull.edu.es (A.C.D.); asantanm@ull.edu.es (Á.S.M.); bsocasro@ull.edu.es (B.S.-R.); avherrer@ull.edu.es (A.V.H.H.)

**Keywords:** dispersive liquid–liquid microextraction (DLLME), umbrella review, systematic review, methodological quality (AMSTAR 2), green analytical chemistry

## Abstract

Over the past two decades, dispersive liquid–liquid microextraction (DLLME) has evolved from an emerging concept into a widely adopted approach within sustainable sample preparation. In parallel, a substantial body of review literature has accumulated, highlighting diverse applications and methodological developments. This umbrella review provides a structured synthesis of 59 review and systematic review articles published between 2006 and 2025, with the aim of examining how the review literature itself has shaped current understanding of DLLME. Methodological quality was appraised using the AMSTAR 2 framework, revealing considerable variability in review design and reporting practices. Key elements such as the transparent reporting of pre-defined review methods, fully reproducible search strategies, and structured assessments of bias were not routinely reported, and the majority of reviews were classified as critically low according to AMSTAR 2 criteria. To contextualize these findings, evidence redundancy was examined through structured overlap analysis, yielding a very low Corrected Covered Area (CCA = 0.0188), which indicates that existing reviews largely address complementary rather than repetitive aspects of DLLME. Thematic synthesis identified three dominant domains: methodological and mechanistic developments, green and sustainable extraction strategies, and application-driven advances in environmental and pharmaceutical analysis. Together, these findings provide a structured basis for improving future review design, evaluation, and editorial assessment in analytical chemistry, supporting more transparent, reproducible, and methodologically aligned evidence synthesis.

## 1. Introduction

Sample preparation has long been recognized as a defining stage in analytical chemistry, often exerting a decisive influence on the reliability, comparability, and interpretability of analytical results. Among modern sample preparation strategies, liquid-phase microextraction (LPME) techniques have emerged as an important family of miniaturized extraction approaches characterized by low solvent consumption, operational simplicity, and compatibility with green analytical chemistry principles. Representative LPME techniques include single-drop microextraction (SDME), hollow-fiber liquid-phase microextraction (HF-LPME), emulsification-based microextraction approaches, and dispersive liquid–liquid microextraction (DLLME), each offering distinct advantages depending on the analytical application and matrix complexity. Within this context, DLLME, introduced by Asadi and co-workers in 2006 [1], rapidly attracted attention as a practical and efficient alternative to conventional extraction approaches. Its combination of simplicity, short extraction times, low solvent consumption, and compatibility with miniaturization positioned DLLME as a particularly attractive strategy at a time when green analytical chemistry (GAC) principles were gaining increasing prominence.

Over the subsequent two decades, DLLME has undergone continuous methodological diversification. Innovations in extraction solvents—including ionic liquids, deep eutectic solvents, and supramolecular systems—have expanded the technique’s selectivity and environmental compatibility. Parallel developments in dispersion strategies, automation, and coupling with chromatographic and spectrometric techniques have further broadened its analytical scope [2]. As DLLME matured from an emerging concept into a widely applied sample-preparation platform, the volume of published primary studies and, consequently, review articles grew rapidly across environmental, food, biological, pharmaceutical, and industrial domains.

This rapid expansion of the field has been accompanied by a similarly rapid growth in review literature. While these reviews have played an essential role in consolidating knowledge and guiding application-driven research, their diversity also reflects heterogeneous practices in review design, reporting, and evidence synthesis. Such heterogeneity is not unexpected in a dynamic and evolving research area; however, it can complicate efforts to obtain a coherent and methodologically consistent overview of the evidence, particularly as expectations for transparency, reproducibility, and rigor in evidence synthesis continue to evolve.

As analytical chemistry moves toward greater methodological standardization and evidence-based decision-making, the maturation of the field brings with it new expectations regarding how review articles are designed, conducted, and interpreted. In this setting, questions naturally arise about the extent to which existing DLLME-focused reviews align with contemporary standards for evidence synthesis, and how differences in review methodology may influence the conclusions drawn about the technique’s development and applications.

An umbrella review provides a structured framework to address these questions. Positioned at the highest level of evidence synthesis, this approach enables a comparative evaluation of review articles themselves, allowing methodological practices, thematic emphases, and evidence structures to be examined collectively rather than in isolation [3,4]. For a rapidly developing technique such as DLLME, this perspective offers an opportunity not to reassess the technique per se, but to understand how the review literature has evolved alongside the field.

Accordingly, the present umbrella review was designed to systematically collect and evaluate all published reviews on DLLME, encompassing narrative, critical, and systematic formats. Specifically, this study aims to (i) map the evolution of DLLME applications across major analytical domains; (ii) identify recurring methodological and technological trends reported in the review literature; (iii) examine review design and reporting practices using the AMSTAR 2 framework; (iv) assess the structure and overlap of the underlying evidence base; and (v) explore how DLLME developments reported in reviews align with the broader principles of green and sustainable sample preparation.

In the context of this study, transparency refers to the explicit and reproducible reporting of review objectives, literature search procedures, eligibility criteria, evidence-selection pathways, and methodological decision-making processes that allow readers to understand, evaluate, and potentially reproduce the review workflow.

By situating these findings within the broader context of an evolving analytical field, this work seeks to support more coherent, transparent, and methodologically aligned review practices. Ultimately, the goal is not to judge past contributions, but to contribute constructively to the ongoing maturation of evidence synthesis in analytical chemistry.

## 2. Methods

This umbrella review was designed to provide a structured overview of how review literature on DLLME has evolved, with particular attention to methodological practices, thematic coverage, and evidence structure. This study is designed as an umbrella review (review of reviews) conducted using systematic and transparent literature identification, screening, and reporting procedures informed by PRISMA 2020 guidelines [5], adapted for umbrella review design. The study protocol was registered on the Open Science Framework (OSF) [6], and the review was conducted in accordance with the JBI Manual for Evidence Synthesis and the PRISMA guidance for umbrella reviews [5], ensuring transparency and reproducibility throughout the process.

### 2.1. Eligibility Criteria

Only systematic reviews (SRs), meta-analyses, narrative reviews, critical reviews, scoping reviews, and book chapters—focusing exclusively on DLLME or its derivatives and addressing applications or methodological advancements were eligible for inclusion. Reviews that covered multiple microextraction techniques where DLLME was only a minor component were excluded to maintain focus and prevent bias in data extraction. Primary research articles, conference reports, letters, and reviews focusing solely on other techniques were also excluded. The inclusion period ranged from 2006 (the year DLLME was first introduced) to 18 May 2025, with no language restrictions applied.

### 2.2. Information Sources and Search Strategy

A comprehensive search was conducted in Scopus, Web of Science (Core Collection), and PubMed. The search strategy combined keywords related to DLLME (“*Dispersive Liquid–Liquid Microextraction*”) with terms associated with review articles (“*review*,” “*systematic review*,” “*meta-analysis*,” “*overview*,” “*critical review*,” “*trends*,” “*advances*”). Detailed queries for each database and search tables are provided in Supplementary File S1. The initial search was conducted on 18 May 2025, and the final update was completed on 7 August 2025.

### 2.3. Study Selection Process

**Scheme 1:**
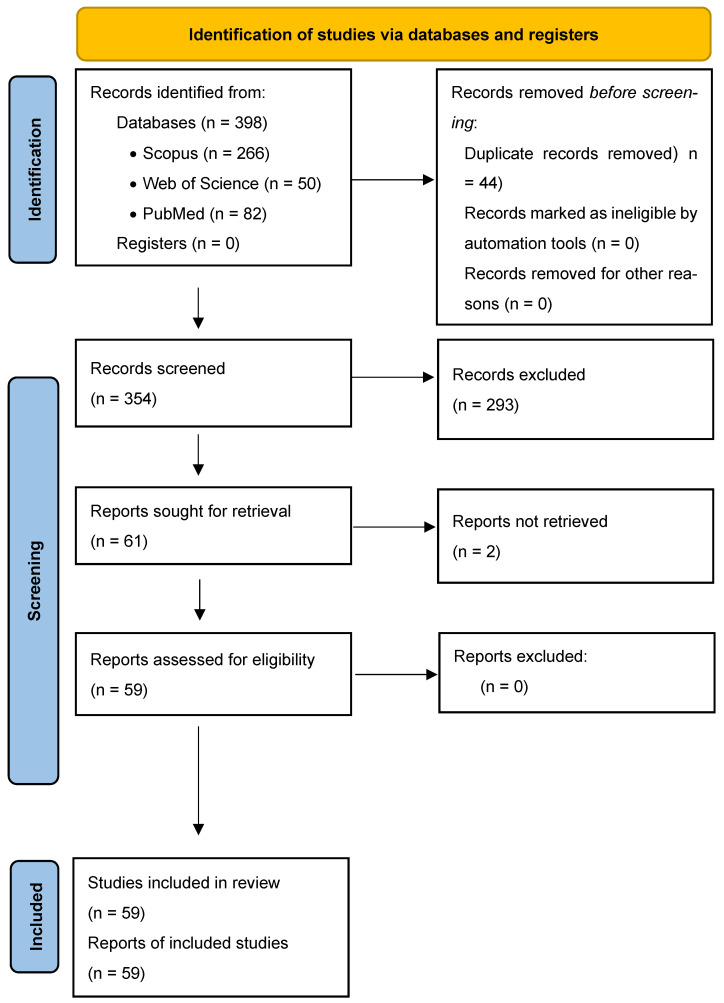
PRISMA 2020 Flow Diagram for the Selection Process of Systematic Reviews and Meta-analyses Related to Dispersive Liquid–Liquid Microextraction (DLLME). Scheme 2020. statement: an updated guideline for reporting systematic reviews. *BMJ*. 2021;372:n71. doi: 10.1136/bmj.n71 [7]. This work is licensed under a Creative Commons Attribution 4.0 International (CC BY 4.0) license.

After duplicate removal, the remaining records were independently screened by reviewers working in pairs. Titles and abstracts were assessed in the first stage, followed by full-text evaluation when necessary. For articles not accessible through electronic databases, the corresponding authors were contacted. Any disagreements between reviewers were resolved through discussion or, when required, consultation with a third reviewer. The overall study selection process is summarized in the PRISMA flow diagram (Scheme 1).

### 2.4. Data Extraction

Data extraction was performed independently by two reviewers using a standardized form (see Supplementary File S2). For non-English sources (e.g., Chinese), translations were generated using AI-assisted tools (ChatGPT-4, desktop version) and subsequently validated by human review to ensure accuracy. Extracted items included bibliographic information, objectives and research questions, scope and timeframe, methodology, results and limitations, future recommendations, methodological quality assessed with AMSTAR 2, application domains, analytes, innovations, and key challenges. All data and supplementary forms were securely stored and are available from the corresponding author upon reasonable request.

### 2.5. Methodological Quality Assessment

The methodological characteristics of the included reviews were examined using the AMSTAR 2 tool [8], originally developed to appraise SRs in clinical and health-related research. In the context of the present study, AMSTAR 2 was applied not as a punitive benchmark, but as a structured analytical framework to explore reporting practices, methodological clarity, and reproducibility within analytical chemistry review literature.

Because several AMSTAR 2 items were designed for intervention-based clinical evidence synthesis, direct application to experimental analytical chemistry reviews may be inappropriate without contextual interpretation. Therefore, the framework was operationally adapted to reflect the methodological characteristics of analytical sciences, particularly sample preparation research. These adaptations included contextual reinterpretation of concepts related to risk of bias, analytical reproducibility, validation practices, matrix effects, optimization strategies, and the limited role of meta-analysis in DLLME-focused reviews.

Particular attention was given to AMSTAR 2 items associated with review planning, evidence identification, study selection, and interpretation, as these elements are increasingly relevant to transparent evidence synthesis across scientific disciplines. Items directly linked to quantitative meta-analysis (Items 11, 12, and 15) were considered not applicable in most cases, reflecting prevailing practices within DLLME review literature rather than methodological omission.

To avoid binary interpretations, item-level assessments were used to describe prevailing methodological patterns rather than to rank or penalize individual reviews. Overall confidence classifications were derived following the AMSTAR 2 guidance and are reported to provide a field-level overview of methodological tendencies, not to evaluate the scientific merit of individual contributions.

The methodological principles discussed throughout this work were also applied, where appropriate, to the design and reporting structure of the present umbrella review itself, including protocol registration, systematic study selection, overlap analysis, and open-access availability of supporting materials.

Detailed calibration rules, contextual adaptations, and item-level assessments are provided in the Supplementary Information (Files S3, S3.1, S3.2 and S4).

### 2.6. Assessment of Primary Study Overlap

To evaluate the degree of overlap among the primary studies included across the final set of SRs, the DOIs of the original primary studies were systematically extracted and utilized as unique identifiers.

In this study, a custom-developed overlap assessment tool was employed. The tool generated an N × N overlap matrix based on the Jaccard similarity index. Subsequently, the overall degree of evidence duplication was quantitatively assessed using the Mean Pairwise Overlap Percentage and the Corrected Covered Area (CCA), which were used to interpret the magnitude of the overlap. Finally, the tool identified the core studies.

The custom overlap assessment tool (HTML format) is directly accessible to readers, allowing for instant replication of the analysis in any web browser via the following executable link: https://hakimfaraji.github.io/Overlap_Analysis_Tool/ accessed on 14 December 2025. The source code for the tool and the raw input and output data (including the N × N overlap matrix and the calculated CCA values in CSV format) are archived in the OSF repository associated with this project, https://osf.io/mxspq/overview accessed on 30 November 2025 [6]. These data are also provided for direct access in Supplementary File S5.1–S5.3.

### 2.7. Synthesis of Findings

Data synthesis was conducted using a narrative and thematic approach. Initial categorization included applications (environmental, food, biological, pharmaceutical/cosmetic, industrial/miscellaneous) and methodological advancements (alternative solvents, automation, miniaturization, integration with other techniques, and process optimization). The contribution of each study to green chemistry, energy consumption, and waste generation was also assessed. Reviews demonstrating more comprehensive reporting and clearer methodological description were given greater interpretative weight in the synthesis. Individual data, major themes, prevailing trends, and knowledge gaps are presented in tables and text throughout the manuscript.

### 2.8. Stakeholder Engagement

Direct stakeholder involvement (industry representatives, policymakers, or other experts) in the design or execution of this umbrella review was not implemented. However, the perspectives and needs of stakeholders were considered during the interpretation of results and the formulation of recommendations for future research.

## 3. Results

### 3.1. Search and Study Selection Results

A systematic and comprehensive search of the Scopus, Web of Science, and PubMed databases, conducted on 18 May 2025, retrieved a total of 398 records (see Supplementary File S6). After importing the data into EndNote and removing duplicates, 354 unique records remained for initial screening. The first-stage screening, involving independent evaluation of titles and abstracts by two reviewers, led to the exclusion of 293 studies (82.8%) based on predefined eligibility criteria. Subsequently, the full texts of 61 SRs focusing on the DLLME technique were thoroughly assessed. Of these, 2 reviews did not fully meet the inclusion criteria, leaving 59 SRs for inclusion in this umbrella review [2,9,10,11,12,13,14,15,16,17,18,19,20,21,22,23,24,25,26,27,28,29,30,31,32,33,34,35,36,37,38,39,40,41,42,43,44,45,46,47,48,49,50,51,52,53,54,55,56,57,58,59,60,61,62,63,64,65,66]. The stepwise study selection process is clearly presented in the PRISMA flow diagram (Scheme 1). Importantly, no studies were excluded due to language or full-text availability, which supports the comprehensiveness and robustness of this review.

### 3.2. Characteristics of Included Systematic Reviews

In total, 59 SRs met the inclusion criteria and were included in this umbrella review. The main descriptive characteristics of each review, including publication year, first author, country of affiliation, journal, and thematic focus, are summarized in Table 1.

**Figure 1:**
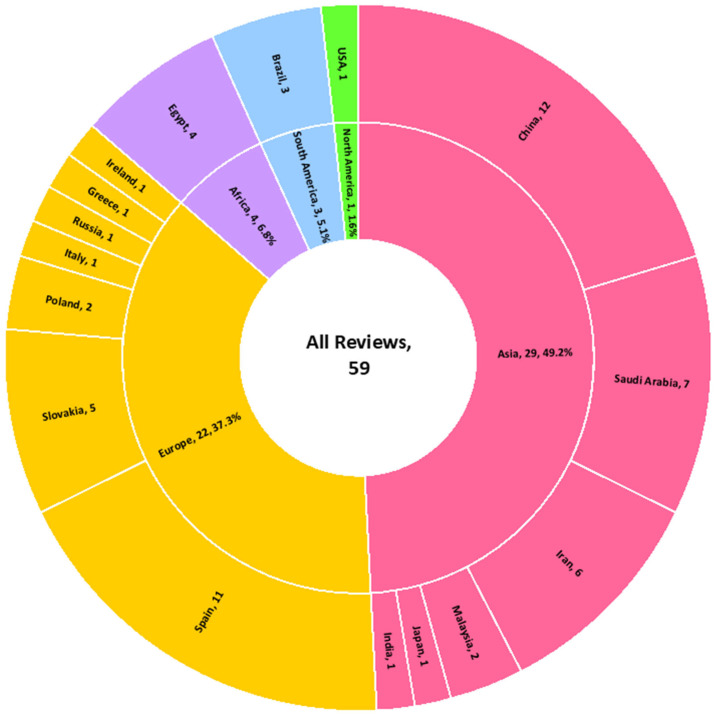
Geographical Distribution of Included Systematic Reviews by First Author’s Affiliation Country (*n* = 59).

The temporal distribution of the included SRs indicates a strong emphasis on recent years, with more than 64% of the reviews (N = 38) published between 2016 and 2025 (Table 1). This pattern reflects the growing scientific interest in DLLME and its continued methodological development over the last decade. From a geographical perspective, first authors were predominantly affiliated with institutions in China (20.3%), Spain (18.6%), Saudi Arabia (11.9%), and Iran (10.2%), which together accounted for 61.0% of all included publications (Supplementary File S1, Table S1 and Figure 1). Among the 59 SRs, 56 were published as journal articles and three as book chapters. These publications were distributed across 30 specialized journals, with TrAC-Trends Anal. Chem. contributing the largest share (11 reviews, 18.6%), followed by Talanta, J. Chromatogr. A, and Microchem. J., each publishing four reviews (Table 1).

Thematic analysis (Table 1) revealed three dominant application areas. Environmental analysis represented the largest share of publications, accounting for 42.4% of the included reviews, highlighting the strong relevance of DLLME for trace-level pollutant monitoring in complex environmental matrices. Reviews focusing on the development of green solvents, including ionic liquids and deep eutectic solvents, accounted for 20.3% of the literature, reflecting the growing alignment of DLLME with green analytical chemistry principles. Applications in food and agricultural matrices constituted 18.6% of the reviews, underscoring the importance of DLLME in food safety and quality control. In addition, methodological aspects such as automation, miniaturization, and device integration were addressed in 10.2% of the reviews, while biological and pharmaceutical applications represented 8.5%.

From a sustainability perspective, 28 SRs (47.5%) explicitly addressed green sample preparation (GSP), with emphasis on innovative solvent systems, reduction in solvent consumption, waste minimization, and improved energy efficiency. The number of primary studies included in individual SRs varied widely, ranging from 6 to 1541, with a mean of 99.5 and a median of 21.5, reflecting substantial heterogeneity in review scope and depth. Notably, almost all reviews relied on narrative or descriptive synthesis approaches (58 SRs; 98.3%), with only one review attempting a more structured analytical framework.

### 3.3. AMSTAR 2 Methodological Quality Results

The methodological characteristics of the 59 included DLLME-focused SRs were examined using the AMSTAR 2 framework, which comprises 16 items spanning seven critical domains related to transparency, planning, evidence identification, and interpretation. The results are presented to describe prevailing reporting and synthesis practices within the review literature, rather than to evaluate the scientific merit of individual contributions.

Based on the AMSTAR 2 decision criteria, none of the included SRs were classified as having high, moderate, or low overall confidence. One review (1.7%) approached a low-confidence classification [12], while the remaining 58 SRs (98.3%) were classified as critically low according to AMSTAR 2 domains, reflecting the presence of multiple critical items that were not routinely addressed. Item-level assessments and the rationale for overall classifications are provided in Supplementary File S4.

Analysis of critical AMSTAR 2 items revealed consistent patterns across the review literature. For Item 2 (pre-definition and structured reporting of review methods), explicit reporting of a pre-defined review protocol—either through formal registration or clear description of review questions, eligibility criteria, and methodological steps within the article—was not observed in the reviewed literature, with only one SR briefly outlining its research objectives [12]. For Item 4 (comprehensive literature search), reproducible and fully documented search strategies were not routinely reported. Two SRs (3.4%) mentioned the databases consulted [44,45], and three SRs (5.1%) reported selected search keywords [43,45,63], while explicit discussion of potential selection limitations was not observed. Regarding Item 7 (justification for excluded studies), documentation of excluded studies and reasons for exclusion was not commonly provided, with one exception [53]. For Item 9 (risk of bias in primary studies), formal risk-of-bias assessment was not routinely incorporated. A limited number of SRs referred to methodological elements such as Design of Experiments (DOE) [12,17,30,39,43] or validation studies [12,17], while several reviews mentioned potential analytical sources of bias—such as matrix interferences or lack of reference materials—without structured evaluation [12,15,17,21,22,26,30,43,65].

Assessment of non-critical AMSTAR 2 items showed more heterogeneous practices. For Item 1 (PICO structure), 35 SRs (59.3%) explicitly defined research questions encompassing all PICO components, while 21 SRs (35.6%) addressed at least two components; three SRs (5.1%) did not clearly define PICO elements [13,16,23]. Items related to duplicate study selection and data extraction (Items 3, 5, and 6) were partially addressed in 29 SRs (49.2%), although parallel extraction by independent reviewers was not reported. Descriptions of included studies (Item 8) varied in detail, ranging from limited (45.8%) to basic but sufficient (50.8%) reporting [14,52]. Funding sources of included studies (Item 10) were not reported. Items related to meta-analysis and publication bias (Items 11, 12, and 15) were not applicable, as quantitative synthesis was not conducted in the included SRs. Consideration of heterogeneity (Item 14) was reported in 33 SRs (55.9%), while 26 SRs (44.1%) did not explicitly address heterogeneity. Conflicts of interest (Item 16) were reported in 40 SRs (67.8%), whereas 19 SRs (32.2%) did not include such statements.

Taken together, these findings indicate that the included DLLME reviews share common structural characteristics in how evidence is planned, identified, and interpreted. These patterns reflect prevailing review-writing conventions in the field rather than isolated methodological oversights.

### 3.4. Primary Study Overlap Results

The overlap of primary studies across the 59 included DLLME SRs was analyzed to assess duplication and coverage within the evidence base.

A total of 5639 references to primary studies were aggregated. After removing duplicates, 2716 unique primary studies were identified, indicating that 52.2% of references were repeated (Supplementary File S5.1). The Jaccard (Commonality) Index, calculated for pairwise SRs overlap, yielded an overall value of 0.0186, reflecting minimal average pairwise overlap (~5.5%).

**Figure 2:**
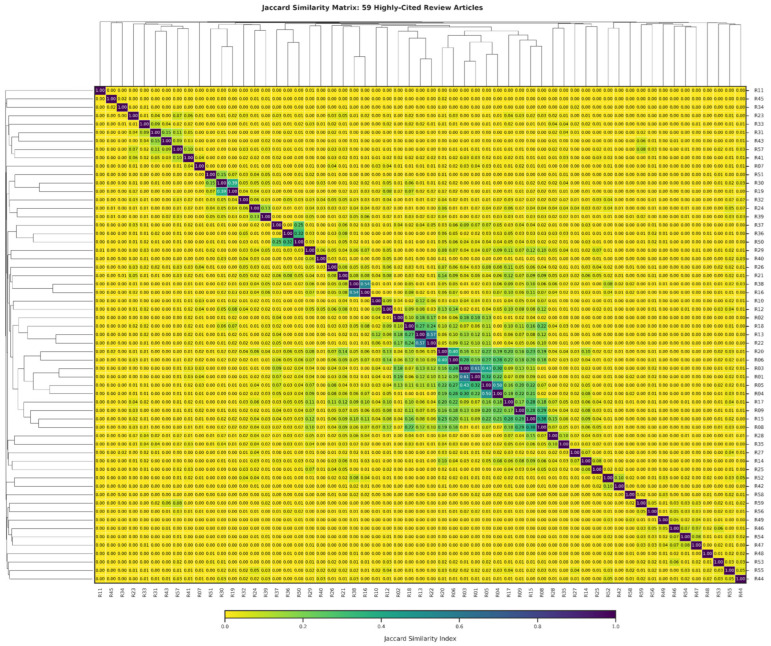
Heatmap Visualization of Pairwise Overlap Among Included SRs. The codes used for the axes (R01–R59) correspond to the unique identifiers assigned to each SR, as detailed in Table 1.

The heatmap illustrates the degree of overlap in primary studies between all pairs of included SRs, calculated based on the Jaccard similarity index (Figure 2). Darker colors indicate a higher proportion of shared primary studies. The analysis confirms that duplication is generally sparse and scattered, with the majority of overlaps occurring only across two to four SRs.

Despite limited pairwise overlap, 1084 primary studies were cited in two or more SRs, representing 39.8% of all unique primary studies. Table S2 in Supplementary File S1 lists the fifteen most frequently cited core primary studies, highlighting their crucial role as the foundation of the evidence base on DLLME. Notably, the original DLLME development study [1] was cited in 42.4% of SRs, highlighting its foundational role. Importantly, these repeated citations reflect inclusion as eligible primary studies rather than mere historical references, indicating strong consensus on their methodological significance.

## 4. Discussion

### 4.1. Introduction and Overall Evidence Landscape

The descriptive analysis of the included SRs provides more than a quantitative snapshot; it offers insight into how the DLLME research landscape has evolved alongside the rapid expansion of the technique itself (Table 1, Figure 1 and Figure 2). The appearance of the first review article in 2008—less than two years after the original introduction of DLLME—reflects the immediate scientific interest in consolidating emerging evidence [9]. At that early stage, review articles were necessarily based on a limited pool of primary studies, underscoring the exploratory role of early evidence synthesis in a fast-developing field.

Notably, 67% of the SRs were published in first-quartile (Q1) journals according to the Web of Science (JCR) classification. This distribution highlights the high visibility and perceived relevance of DLLME-focused reviews within analytical chemistry, rather than serving as a direct proxy for methodological uniformity. As such, these reviews have played a substantial role in shaping research directions and community expectations.

The pronounced increase in review publications after 2016—accounting for more than 64% of all SRs—suggests that DLLME has reached a stage of methodological maturation (Table 1). At this stage, review writing increasingly serves not only to summarize applications but also to guide best practices, compare methodological variants, and support informed decision-making. The geographical concentration of reviews in China, Spain, Saudi Arabia, and Iran (61%) reflects the presence of active research hubs that have contributed extensively to both methodological development and evidence consolidation (Figure 1). Several institutions appear repeatedly across the review landscape, particularly research groups affiliated with the University of La Laguna (Spain), Chinese Academy-related institutions, and major analytical chemistry centers in Iran and Saudi Arabia. These groups have contributed not only to methodological development of DLLME, but also to the consolidation and dissemination of knowledge through influential review articles.

The observed regional distribution—dominated by Asia (49.2%) and Europe (37.3%)—likely reflects differences in research priorities, infrastructure, and sustainability-driven innovation pathways rather than qualitative distinctions in scientific rigor (Table S1 and Figure 1). In regions where low-cost, rapid, and environmentally aligned analytical solutions are particularly valued, DLLME has emerged as a focal technique for both application and review-based synthesis.

Thematic patterns further reinforce this interpretation. The predominance of environmental analysis (42.4%) and the strong emphasis on green solvent systems (20.3%) indicate sustained demand for DLLME in complex matrices and sustainability-oriented contexts (Table 1). Concurrent attention to automation, miniaturization, and system integration reflects a broader transition from exploratory method development toward performance optimization, reproducibility, and scalability.

Taken together, these patterns suggest that the DLLME review literature has expanded in parallel with the technique’s scientific maturation. This evolution creates a timely opportunity to reflect on how review-writing practices can continue to adapt, ensuring that evidence synthesis remains aligned with the growing complexity, diversity, and expectations of the field.

### 4.2. Methodological Quality of Existing Reviews: Insights from an Adapted AMSTAR 2 Perspective

The assessment of methodological practices across the included reviews, conducted using an adapted application of the AMSTAR 2 framework, provides important insights into how evidence synthesis has evolved alongside the rapid expansion of DLLME research. It is important to emphasize that AMSTAR 2—originally developed for clinical SRs—was applied here not as a punitive benchmark, but as a structured and transparent lens to explore reporting, reproducibility, and decision-making practices within analytical chemistry review.

Within this adapted context, all 59 reviews were classified as having critically low confidence according to AMSTAR 2 criteria, reflecting the presence of multiple critical domains that were not routinely addressed. Rather than indicating isolated methodological oversights, this pattern highlights prevailing review-writing conventions that emerged during periods of rapid methodological innovation, when formal standards for evidence synthesis in experimental analytical chemistry were still evolving literature (see Supplementary File S4).

One prominent observation concerns the a priori definition and transparent reporting of review methods (Item 2). Explicit statements indicating the use of a pre-defined review protocol—either through formal registration or detailed methodological description within the article—were not commonly reported, and key elements such as explicit inclusion criteria, predefined analytical objectives, or deviation management strategies were often described only implicitly. In fast-moving methodological fields such as DLLME, reviews have traditionally been conceived as flexible narrative syntheses, which may partly explain the limited uptake of formal protocol-based approaches.

Similarly, with respect to literature search strategies (Item 4), reproducible and fully documented search procedures were not routinely reported. While most reviews aimed to provide comprehensive coverage of the field, the absence of detailed search documentation limits reproducibility and makes it difficult to assess the completeness of evidence capture. This finding reflects historical norms in analytical chemistry review writing, where methodological clarity in search strategy has not always been considered essential.

The documentation of excluded studies (Item 7) was also infrequently reported. In many cases, study selection decisions were described at a conceptual level rather than through explicit exclusion lists. Although this practice aligns with traditional narrative review formats, it constrains the ability of readers to independently assess selection pathways or reproduce the review process.

A particularly relevant aspect for experimental sciences relates to the assessment of bias and data reliability in primary studies (Item 9). Formal risk-of-bias assessment, as defined in clinical research, was not routinely incorporated. Instead, some reviews discussed analytical challenges—such as matrix effects, validation practices, or optimization strategies—without framing these considerations within a systematic bias assessment framework. This highlights a conceptual gap rather than an absence of critical awareness, underscoring the need for bias-assessment models that better reflect the epistemic structure of analytical method development.

Consistent with this observation, the impact of data quality on result interpretation (Item 13) was rarely addressed explicitly. Conclusions were typically drawn from aggregated findings without structured reflection on how methodological variability among primary studies might influence synthesis outcomes. Again, this pattern mirrors long-standing conventions in method-oriented reviews rather than deliberate neglect.

Items related to meta-analysis and publication bias (Items 11, 12, and 15) were classified as not applicable, as none of the included reviews pursued quantitative synthesis. While meta-analysis remains uncommon in DLLME research due to methodological heterogeneity, its absence limits the capacity to quantitatively compare performance trends or methodological advances across studies.

Taken together, these findings should be interpreted as indicators of a field in maturation. The rapid growth of DLLME has stimulated an equally rapid expansion of review literature, often preceding the widespread adoption of formal evidence-synthesis standards. From this perspective, the adapted AMSTAR 2 analysis serves less as a judgment of past work and more as a diagnostic tool, identifying areas where emerging expectations for structured reporting and reproducibility could be progressively integrated into future review practice.

Importantly, these patterns reflect structural and historical characteristics of DLLME review writing, rather than individual shortcomings. As expectations for evidence synthesis continue to evolve within analytical chemistry, the integration of selected SR principles—appropriately adapted to experimental contexts—offers a pathway toward more transparent, reproducible, and analytically robust reviews.

### 4.3. Interpretation of Findings in Light of Primary Study Overlap

The analysis of primary study overlap across the 59 included reviews provides a nuanced view of how evidence synthesis has developed within the DLLME field. The overall Jaccard Index (0.0186), corresponding to a mean pairwise overlap of approximately 5.5%, indicates a remarkably low level of redundancy among reviews. As illustrated in the overlap heatmap (Figure 2), this dispersed pattern suggests that individual reviews have generally concentrated on distinct methodological themes or application domains rather than reiterating previously synthesized content.

This low overlap reflects the inherently modular evolution of DLLME research. Reviews have typically focused on specific dimensions such as solvent innovation, optimization of extraction parameters (e.g., pH, ionic strength, dispersants), or targeted applications in environmental, food, or biological matrices. From this perspective, the observed dispersion represents thematic specialization rather than fragmentation, underscoring the breadth and adaptability of DLLME as an analytical approach.

At the same time, the identification of 1084 primary studies cited in two or more reviews—accounting for 39.8% of unique sources—highlights the presence of a shared foundational literature. Seminal contributions, including the original work by Rezaee and co-workers, function as common reference points across diverse reviews, indicating broad consensus regarding the methodological origins and core performance characteristics of DLLME [1].

Together, these patterns suggest that existing reviews offer complementary rather than repetitive perspectives. The limited overlap emphasizes the value of integrative synthesis across subfields, while the concentration around core studies provides a stable basis for evaluating how consistently foundational evidence has been interpreted. From an evidence-consumption standpoint, these findings encourage readers to view individual reviews as domain-specific lenses rather than comprehensive representations of the entire DLLME landscape.

### 4.4. Synthesized Findings: Key Trends in Applications and Methodological Advances of DLLME

The synthesis of findings across the included reviews illustrates that DLLME has evolved along multiple, interconnected trajectories rather than following a single linear path. Collectively, the review literature portrays DLLME as a flexible analytical platform whose development has been shaped by application-driven demands, methodological refinement, and sustainability considerations.

Environmental analysis remains the most frequently addressed application domain, reflecting the technique’s suitability for trace-level determination of pollutants in complex matrices. At the same time, substantial growth in food, agricultural, and biological applications highlights an expanding demand for rapid, sensitive, and low-volume sample preparation strategies in regulated and high-impact analytical contexts.

The review literature also demonstrates the broad compatibility of DLLME with a wide range of analytical instrumentation. Gas chromatography (GC) and high-performance liquid chromatography (HPLC) were the most frequently reported platforms, often coupled with mass spectrometry (MS), flame ionization detection (FID), ultraviolet detection (UV), and atomic spectrometric techniques such as AAS, ICP-OES, and ICP-MS. This instrumental versatility has played a major role in the widespread adoption of DLLME across environmental, pharmaceutical, food, and biological applications.

Across application areas, reviews consistently report broad adaptability with respect to analyte classes and matrix complexity. The increasing focus on emerging contaminants—such as pharmaceuticals and personal care products—illustrates how DLLME continues to respond to evolving analytical challenges rather than being confined to legacy applications.

Methodological innovation has largely centered on improving enrichment efficiency, phase separation, and workflow integration. Variants such as DLLME-SFO and hybrid extraction approaches exemplify efforts to enhance robustness while reducing operational complexity. Notably, the growing emphasis on automation and flow-based configurations reflects a strategic shift toward higher throughput, reduced operator dependence, and improved reproducibility.

Alignment with green analytical chemistry principles represents a defining feature of recent DLLME development. Reviews increasingly highlight the use of ionic liquids and deep eutectic solvents as alternatives to conventional organic solvents. At the same time, the literature acknowledges unresolved questions regarding long-term toxicity, biodegradability, and life-cycle impacts, suggesting that sustainability assessment must extend beyond solvent substitution alone.

Overall, the synthesized evidence indicates that DLLME has transitioned from a niche microextraction technique to a versatile, application-responsive platform. Continued progress will depend on integrating methodological innovation with standardized evaluation practices and more comprehensive sustainability metrics.

### 4.5. Strengths and Limitations of the Present Umbrella Review

This umbrella review was designed to provide a comprehensive, structured overview of the DLLME review literature, and its strengths and limitations should be interpreted within this context.

A key strength lies in the breadth of the evidence base considered. By synthesizing findings from 59 reviews spanning nearly two decades, the study captures the diversity of DLLME applications and methodological developments across multiple analytical domains. The incorporation of quantitative overlap analysis further strengthens interpretation by clarifying evidence independence and contextualizing repeated citation patterns.

The adapted use of the AMSTAR 2 framework represents another important strength. Rather than serving as a rigid evaluative checklist, it functioned as a calibration tool to systematically explore traceability of evidence and reproducibility practices in analytical review writing, thereby supporting a more nuanced interpretation of synthesized findings.

Several limitations should also be acknowledged. The heterogeneity of review designs, analytical targets, and outcome metrics limited the feasibility of quantitative synthesis, necessitating a primarily narrative and thematic approach. In addition, variability in reporting practices constrained deeper methodological comparison across reviews. As with most literature-based syntheses, incomplete coverage of gray literature may also have influenced the evidence landscape captured.

Despite these limitations, the present umbrella review provides a coherent and integrative perspective on the DLLME literature. By combining methodological appraisal, overlap analysis, and thematic synthesis, it offers a transparent reference framework that can inform future review design, editorial evaluation, and evidence-informed methodological development in analytical chemistry.

## 5. Conclusions and Forward-Looking Recommendations

This umbrella review synthesized evidence from 59 narrative and SRs addressing DLLME, with the objective of mapping thematic coverage, developmental trajectories, and methodological practices within the review literature itself rather than reassessing DLLME as an analytical technique.

### 5.1. Thematic Coverage and Developmental Trends

Thematic analysis indicates that DLLME-related reviews have predominantly addressed environmental, food, and biological matrices. Among the included reviews, 38 (65%) focused on environmental applications, 25 (43%) on food-related matrices, and 18 (30%) on biological and clinical samples. This distribution reflects both the versatility of DLLME and the tendency of individual reviews to concentrate on specific analytical contexts, such as solvent innovation, optimization of extraction parameters (e.g., pH, salt concentration, dispersants), or matrix-specific challenges.

Temporal trends further suggest a gradual shift toward emerging analytical priorities, including monitoring of emerging contaminants and the adoption of greener solvent systems. Together, these patterns highlight the adaptive nature of DLLME research and the capacity of the review literature to respond to evolving scientific and regulatory demands.

### 5.2. Evidence Reliability and Methodological Maturity

Application of the AMSTAR 2 framework—used here as an exploratory and adaptive methodological lens—indicates that current review-writing practices in the DLLME field reflect heterogeneous and evolving standards. Across the included reviews, explicit reporting of pre-defined review protocols—either through formal registration or transparent description of review methods—was not commonly observed; search strategies were often insufficiently documented to allow full reproducibility, and formal assessment of bias in primary studies was not routinely incorporated.

Rather than representing isolated shortcomings, these patterns appear to reflect the historical context in which many DLLME reviews were produced, during a period when methodological expectations for evidence synthesis in analytical chemistry were still emerging. Consequently, the findings of existing reviews should be interpreted with appropriate contextual awareness, particularly when used to inform comparative evaluations or methodological decision-making.

### 5.3. Implications for Evidence Synthesis in Analytical Chemistry

A notable observation is the absence of fully quantitative SRs or meta-analyses within the DLLME review literature. While narrative synthesis has provided valuable conceptual and thematic insight, the limited use of quantitative approaches constrains the ability to formally compare DLLME performance across studies or against alternative techniques. This gap does not diminish the value of existing reviews but highlights an opportunity for methodological expansion as the field continues to mature.

### 5.4. Strategic Recommendations

Based on the synthesized findings, several forward-looking recommendations can be outlined:

- **Capacity building in review methodology.** Greater familiarity with established reporting and synthesis frameworks (e.g., PRISMA, AMSTAR 2) can support more transparent and reproducible review practices tailored to analytical chemistry.
- **Encouragement of quantitative synthesis where feasible.** Incorporating meta-analytical approaches, when supported by data structure and study design, may enhance comparative evaluation of DLLME performance and methodological progress.
- **Contextual adaptation of methodological standards.** General evidence-synthesis frameworks should be thoughtfully adapted to account for DLLME-specific features, including optimization strategies, validation practices, and matrix effects.
- **Strengthening transparency and reproducibility.** Clear articulation of review objectives, eligibility criteria, and search strategies can facilitate reproducibility and improve confidence in synthesized conclusions.
- **Integration of data quality and bias considerations.** Systematic attention to primary study quality and potential sources of bias can further support robust interpretation of review findings.
- **Expansion of umbrella review approaches.** Applying umbrella review methodologies across related areas of analytical chemistry may help clarify evidence structures, identify convergent trends, and guide more targeted methodological development.

Collectively, these recommendations aim to support the continued maturation of review-writing practices in analytical chemistry. By aligning methodological rigor with the evolving complexity of DLLME research, future reviews can play a more effective role in guiding innovation, promoting sustainability, and strengthening the analytical evidence base. Importantly, the present work does not advocate rigid uniformity in review writing, nor does it aim to limit the interpretative or creative dimensions of scholarly reviews. Rather, it proposes that certain core elements of evidence synthesis—such as transparent reporting of review scope, search methodology, and evidence selection—may benefit from greater methodological clarity, while still preserving the diversity of scientific perspectives and narrative styles that characterize analytical chemistry literature.

## Figures and Tables

**Table 1:** Descriptive Characteristics and Thematic Scope of Included Systematic Reviews.

Code ^a^	Article Title	Year	Total Primary Studies	Lead Author	Country	Journal	Core Focus and Scope of Review	Ref.
R01	Developments of dispersive liquid-liquid microextraction technique	2009	39	Zang, X.H.	China	Chin. J. Anal. Chem.	A comprehensive review of DLLME fundamentals, performance parameters, and recent applications in environmental, food, and biological analysis using GC, HPLC, and AAS, highlighting advantages and future trends.	[9]
R02	Recent developments in homogeneous and dispersive liquid-liquid extraction for inorganic elements determination. A review	2009	37	Anthemidis, A.N.	Greece	Talanta	Overview of HLLE and DLLME as sample preparation techniques for inorganic analysis, focusing on the determination of metal ions, metalloids, and organometals, emphasizing miniaturization and automation advancements.	[10]
R03	Separation and preconcentration by dispersive liquid-liquid microextraction procedure: A review	2009	48	Ojeda, C.B.	Spain	Chromatographia	Summary of DLLME applications and methodological developments for the extraction and preconcentration of organic and inorganic compounds, including optimization parameters and coupling with chromatographic and spectrometric techniques.	[11]
R04	Dispersive liquid-liquid microextraction for determination of organic analytes	2010	92	Herrera-Herrera, A.V.	Spain	Trends Anal. Chem.	Applications of DLLME for extracting organic analytes like pesticides and pharmaceuticals from various matrices, examining analytical performance, method development, and the integration of ionic liquids and green solvents.	[12]
R05	Evolution of dispersive liquid-liquid microextraction method	2010	102	Rezaee, M.	Iran	J. Chromatogr. A	Review of DLLME principles, advancements, limitations, and applications in environmental, biological, and food sample analysis, focusing on its coupling with GC, HPLC, AAS, and other analytical instrumentation.	[13]
R06	Dispersive liquid-liquid microextraction	2011	50	Agnieszka, Z.G.	Poland	Trends Anal. Chem.	Summary of recent advances in sample preparation for trace element determination in various food samples (e.g., milk, meat), with a specific focus on DLLME methodology.	[14]
R07	Liquid-phase and dispersive liquid-liquid microextraction techniques with derivatization: Recent applications in bioanalysis	2011	45	Nuhu, A.A.	Saudi Arabia	J. Chromatogr. B	Reviewing the principles and methodological advancements of DLLME and its variations for determining various analytes in clinical, biological, and environmental samples.	[15]
R08	Separation and preconcentration by dispersive liquid-liquid microextraction procedure: Recent applications	2011	167	Ojeda, C.B.	Spain	Chromatographia	Principles, applications, and variations of DLLME, specifically those employing ionic liquids and green solvents, for determining organic and inorganic species in various analytical matrices.	[16]
R09	Dispersive liquid-liquid microextraction	2012	119	Assadi, Y.	Iran	Comprehensive Sampling and Sample Preparation ^b^	Review on integrating ionic liquids and nanomaterials (e.g., nanoparticles) into DLLME methods for the efficient extraction and determination of trace metal ions.	[17]
R10	Recent advances in coupling single-drop and dispersive liquid-liquid microextraction with UV-vis spectrophotometry and related detection techniques	2012	46	Andruch, V.	Slovakia	Microchem. J.	Comprehensive review of recent DLLME developments and modified techniques for the accurate determination of pesticides across various food matrices, including UA-DLLME and solidified floating drop methods.	[18]
R11	Recent advances in dispersive liquid-liquid microextraction for organic compounds analysis in environmental water: A Review	2012	82	Ma, J.	China	Curr. Anal. Chem.	Applications of DLLME and its variants (e.g., UA-DLLME, A-DLLME) for the extraction of various organic pollutants from environmental water samples.	[19]
R12	Recent advances in dispersive liquid-liquid microextraction using organic solvents lighter than water. A review	2012	40	Kocúrová, L.	Slovakia	Microchem. J.	Applications of DLLME and its variants for the separation and preconcentration of trace metals in various analytical matrices, including environmental, biological, and food samples.	[20]
R13	Dispersive liquid-liquid microextraction for chemical speciation and determination of ultra-trace concentrations of metal ions	2013	37	El-Shahawi M.S.	Saudi Arabia–Egypt ^c^	Trends Anal. Chem.	Overview of utilizing nanomaterials as extractants in DLLME for enhanced determination of inorganic species like metals and metalloids, including their classification, synthesis, and application.	[21]
R14	Dispersive liquid-liquid microextraction in food analysis. A critical review Microextraction Techniques	2013	73	Viñas, P.	Spain	Anal. Bioanal. Chem.	Applications of DLLME and its modified techniques for determining pesticides, antibiotics, additives, and trace elements in food and beverage samples.	[22]
R15	Five years of dispersive liquid-liquid microextraction	2013	431	Andruch, V.	Slovakia	Appl. Spectrosc. Rev.	Summarizing the current status and progress of DLLME and its variants for the analysis of various classes of pesticides in environmental and food samples.	[23]
R16	Ionic liquids in dispersive liquid-liquid microextraction	2013	84	Trujillo-Rodríguez, M.J.	Spain	Trends Anal. Chem.	Recent developments in DLLME variants coupled with atomic spectrometry (AAS, ICP-MS, ICP- OES) for the efficient preconcentration and determination of inorganic elements and species.	[24]
R17	Recent development and applications of dispersive liquid-liquid microextraction	2013	147	Yan, H.	China	J. Chromatogr. A	Summarizing the applications, characteristics, and potential of DESs as extraction solvents in various microextraction techniques, notably DLLME, for organic and inorganic analytes.	[25]
R18	The present state of coupling of dispersive liquid-liquid microextraction with atomic absorption spectrometry	2013	88	Andruch, V.	Slovakia	J. Anal. At. Spectrom.	Development of DLLME variants coupled with analytical instruments for the efficient extraction and determination of non-steroidal anti-inflammatory drugs in environmental, food, and biological samples.	[26]
R19	Automated in-syringe dispersive liquid-liquid microextraction	2014	17	Maya, F.	Spain	Trends Anal. Chem.	Utilization of various DLLME modes coupled with HPLC and GC for the effective extraction and determination of antidepressants in biological and environmental matrices.	[27]
R20	Beyond dispersive liquid-liquid microextraction	2014	150	Leong, M.	China	J. Chromatogr. A	Recent advances in DLLME and its modifications for the extraction and determination of various classes of antibiotics across food, environmental, and biological samples.	[28]
R21	Recent developments in dispersive liquid-liquid microextraction Microextraction Techniques	2014	85	Saraji, M.	Iran	Anal. Bioanal.Chem.	Applications of DLLME variants (e.g., USA-DLLME) coupled with GC and HPLC for the determination of phthalate esters in food, environmental, and biological samples.	[29]
R22	The recent developments in dispersive liquid-liquid microextraction for preconcentration and determination of inorganic analytes	2014	26	Al-Saidi, H.M.	Saudi Arabia	J. Saudi Chem. Soc.	Summarizing the classification, characteristics, and application of DESs in DLLME and other microextraction techniques for analyzing organic and inorganic analytes.	[30]
R23	Dispersive liquid-liquid microextraction: Trends in the analysis of biological samples	2015	33	Zuloaga, O.	Spain	Bioanalysis	Applications and methodological advancements of DLLME variants (e.g., HS-DLLME) coupled with chromatographic and spectrometric techniques for mycotoxin analysis in food and feed samples.	[31]
R24	Progress of extraction solvent dispersion strategies for dispersive liquid-liquid microextraction	2015	85	Li, M.J.	China	Chin. J. Anal. Chem.	Review on ionic liquid (IL)-based microextraction methods, specifically IL-DLLME, for the efficient extraction and preconcentration of inorganic species like metals and metalloids in diverse samples.	[32]
R25	Recent advances in dispersive liquid-liquid microextraction for pesticide analysis	2015	56	Ahmad, W.	Saudi Arabia	Trends Anal. Chem.	Applications of DLLME variants coupled with GC and HPLC for the determination of VOCs across environmental, biological, and food samples.	[33]
R26	Application of hollow fiber liquid phase microextraction and dispersive liquid-liquid microextraction techniques in analytical toxicology	2016	93	Sharifi, V.	Iran	J. Food Drug Anal.	Application of DLLME variants coupled with HPLC and spectrometry for the extraction and determination of food colorants and additives in diverse food matrices.	[34]
R27	Dispersive liquid-liquid microextraction in the analysis of milk and dairy products: A review	2016	41	Quigley, A.	Ireland	J. Chem.	Applications and methodological variations in DLLME (e.g., USA-DLLME) coupled with chromatographic and spectrometric techniques for the extraction of phenolic compounds in environmental and food samples.	[35]
R28	Ten years of dispersive liquid–liquid microextraction and derived techniques	2016	1541	Campillo, N.	Spain	Appl. Spectrosc. Rev.	Principles, applications, and methodological advances of DLLME and its modified modes for the extraction of biomolecules (e.g., amino acids, proteins) from biological samples.	[36]
R29	An overview of advances in dispersive liquid–liquid microextraction for the extraction of pesticides and emerging contaminants from environmental samples	2017	110	Primel, E.G.	Brazil	Trends Environ. Anal. Chem.	Applications of DLLME variants coupled with analytical instruments for the efficient extraction and determination of herbicides in environmental and food samples.	[37]
R30	Automation of dispersive liquid–liquid microextraction and related techniques. Approaches based on flow, batch, flow-batch and in-syringe modes	2017	44	Alexovič, M.	Slovakia	Trends Anal. Chem.	Applications of DLLME variants coupled with chromatographic and spectrometric techniques for the extraction and determination of veterinary drugs in food and biological samples.	[38]
R31	Pharmaceutical and biomedical applications of dispersive liquid–liquid microextraction	2017	41	Mansour, F.R.	Egypt	J. Chromatogr. B	Applications of DLLME variants coupled with GC and HPLC for the efficient extraction and determination of PAHs in environmental, food, and biological samples.	[39]
R32	Recent developments of dispersive liquid-liquid microextraction technique	2017	35	Zang, X.	China	Chin. J. Chromatogr.	Applications of DLLME variants coupled with analytical instruments for the extraction and determination of various analytes in cosmetics and personal care products.	[40]
R33	Solidification of floating organic droplet in dispersive liquid-liquid microextraction as a green analytical tool	2017	89	Mansour, F.R.	Egypt	Talanta	Principles, applications, and recent developments of DLLME variants coupled with analytical techniques for the extraction and determination of water-soluble vitamins in diverse matrices.	[41]
R34	A review on extraction solvents in the dispersive liquid-liquid microextraction	2018	65	Tan, Y.H.	Malaysia	Malays. J. Anal. Sci.	Applications of DLLME variants coupled with GC and HPLC for the extraction and determination of essential oils and their constituents in food, herbal, and biological samples.	[42]
R35	Applications and opportunities of experimental design for the dispersive liquid–liquid microextraction method—A review	2018	194	Mousavi, L.	Iran	Talanta	Applications and methodological variations in DLLME coupled with atomic spectrometry for the extraction and determination of metal nanoparticles (MNPs) in environmental and biological samples.	[43]
R36	Dispersive liquid-liquid microextraction based binary extraction techniques prior to chromatographic analysis: A review	2018	71	Sajid, M.	Saudi Arabia	Trends Anal. Chem.	Reviewing the synthesis, characteristics, and application of hydrophobic DESs in DLLME and other microextraction techniques for organic and inorganic analyte analysis.	[44]
R37	Dispersive liquid-liquid microextraction coupled with derivatization: A review of different modes, applications, and green aspects	2018	39	Sajid, M.	Saudi Arabia	Trends Anal. Chem.	Principles and applications of DLLME and its variants for the extraction of drugs of abuse and other forensic analytes from biological and non-biological forensic samples.	[45]
R38	Modern approaches in dispersive liquid-liquid microextraction (DLLME) based on ionic liquids: A review	2018	148	Rykowska, I.	Poland	J. Mol. Liq.	Applications and methodological advancements of DLLME variants coupled with GC and HPLC for the extraction and determination of various classes of pesticides in environmental and food samples.	[46]
R39	Recent trends in replacement of disperser solvent in dispersive liquid-liquid microextraction methods	2018	59	Sereshti, H.	Iran	Sep. Purif. Rev.	Applications of DLLME variants coupled with HPLC and spectrometry for the extraction and determination of dyes and pigments in environmental and food samples.	[47]
R40	Solvent-terminated dispersive liquid-liquid microextraction: a tutorial	2018	20	Mansour, F.R.	Egypt	Anal. Chim. Acta	Summarizing the synthesis, characteristics, and application of supramolecular solvents (SUPRAS) in DLLME and other microextraction techniques for organic and inorganic analyte analysis.	[48]
R41	Application of Dispersive Liquid-Liquid Microextraction in Forensic Toxicological Analysis	2019	24	Yang, Y.	China	J. Forensic Med.	Applications and methodological variations in DLLME variants coupled with HPLC and spectrometry for the extraction and determination of polar organic compounds in diverse samples.	[49]
R42	Ionic liquid dispersive liquid–liquid microextraction for pesticide residue analysis in honey	2019	6	Zheng, Y.Z.	China	J. Apic. Res.	Applications of DLLME variants coupled with HPLC and GC for the extraction and determination of hormones and steroids in biological and environmental samples.	[50]
R43	Advances in dispersive liquid-liquid microextraction and its application to analysis of biological samples	2020	36	Yiwei, S.	China	Chin. J. Chromatogr.	Applications of DLLME variants coupled with GC and HPLC for the extraction and determination of disinfection by-products (DBPs) in water samples.	[51]
R44	Dispersive liquid-liquid microextraction	2020	88	Kokosa, J.M.	United States	Liquid-Phase Extraction ^b^	Applications of DLLME variants coupled with HPLC and GC for the extraction and determination of explosives and their residues in environmental and forensic samples.	[52]
R45	Dispersive Liquid–Liquid Microextraction of Organic Compounds: An Overview of Reviews	2020	69	Dmitrienko, S.G.	Russia	J. Anal. Chem.	Applications and methodological variations in DLLME variants coupled with GC and HPLC for the extraction and determination of illicit drugs in forensic and biological samples.	[53]
R46	Recent Application of Deep Eutectic Solvents as Green Solvent in Dispersive Liquid–Liquid Microextraction of Trace Level Chemical Contaminants in Food and Water	2020	44	Lu, W.	China–USA ^c^	Crit. Rev. Anal. Chem.	Review on the synthesis, characteristics, and application of magnetic nanomaterials in DLLME for the enhanced extraction and determination of organic and inorganic analytes.	[54]
R47	Deep eutectic solvents as promising green solvents in dispersive liquid–liquid microextraction based on solidification of floating organic droplet: Recent applications, challenges and future perspectives	2021	47	El-Deen, A.K.	Japan–Egypt ^c^	Molecules	Recent advances in DLLME and its modifications for the efficient preconcentration and determination of heavy metals (e.g., lead, mercury) in environmental, food, and biological samples.	[55]
R48	Insights into coacervative and dispersive liquid-phase microextraction strategies with hydrophilic media—A review	2021	102	Pacheco-Fernández, I.	Spain	Anal. Chim. Acta	Applications and methodological variations in DLLME coupled with chromatographic and spectrometric techniques for the extraction and determination of various food contaminants.	[56]
R49	Strategies of dispersive liquid-liquid microextraction for coastal zone environmental pollutant determination	2021	37	Wang, Y.	China	J. Chromatogr. A	Principles, applications, and methodological advances of DLLME for the extraction of drugs, poisons, and their metabolites from forensic toxicology samples.	[57]
R50	Dispersive liquid-liquid microextraction: Evolution in design, application areas, and green aspects	2022	46	Sajid, M.	Saudi Arabia	Trends Anal. Chem.	Applications of DLLME variants coupled with GC and HPLC for the extraction and determination of various classes of pesticides in tea samples.	[58]
R51	In-syringe dispersive liquid-liquid microextraction	2022	32	Lemos, V.A.	Brazil	Talanta	Applications and methodological variations in DLLME variants coupled with LC-MS/MS for the extraction and determination of per- and polyfluoroalkyl substances (PFASs) in environmental and biological samples.	[59]
R52	Ionic Liquid-Assisted DLLME and SPME for the Determination of Contaminants in Food Samples	2022	37	Nie, L.	China	Separations	Reviewing the synthesis, characteristics, and application of natural-DESs in DLLME for the extraction and determination of natural products and pharmaceuticals.	[60]
R53	Use of green alternative solvents in dispersive liquid-liquid microextraction: A review	2022	40	Grau, J.	Spain	J. Sep. Sci.	Applications of DLLME variants coupled with HPLC and spectrometry for the extraction and determination of water-soluble and fat-soluble vitamins in various matrices.	[61]
R54	Recent advances in dispersion strategies for dispersive liquid–liquid microextraction from green chemistry perspectives	2023	198	Kamal El-Deen, A.	Egypt	Microchem. J.	Advancements in DLLME using green solvents (e.g., DESs) for the extraction of organic pollutants like pesticides and pharmaceuticals from environmental water and soil samples.	[62]
R55	Advancements in overcoming challenges in dispersive liquid-liquid microextraction: An overview of advanced strategies	2024	63	Faraji, H.	Spain	Trends Anal. Chem.	Comprehensive overview of green DLLME techniques, detailing green solvent selection, dispersion strategies, phase separation, extractant collection, and method automation based on green chemistry.	[2]
R56	Dispersive Liquid–Liquid Micro Extraction: An Analytical Technique Undergoing Continuous Evolution and Development—A Review of the Last 5 Years	2024	54	Notardonato, I.	Italy	Separations	Evolution of DLLME techniques, covering applications in environmental, food, and biological matrices, methodological improvements, combination with other techniques, derivatization, and green solvents.	[63]
R57	Dispersive liquid-liquid microextraction for forensic drug analysis	2025	71	Mudiam, M.K.R.	India	Green Analytical Methods and Miniaturized Sample Preparation Techniques for Forensic Drug Analysis ^b^	Principles, applications, and modifications of DLLME for forensic drug analysis, covering diverse biological matrices (urine, plasma) and analyte classes (drugs of abuse), including UA-DLLME and DES-DLLME variations.	[64]
R58	Ionic liquids in dispersive liquid–liquid microextraction for environmental aqueous samples	2025	26	Amaral, L.R.	Brazil	Microchem. J.	Applications of ionic liquids in DLLME for environmental aqueous samples, detailing physicochemical properties, various method variants, analyte types, and green chemistry aspects.	[65]
R59	Recent Advances in Dispersive Liquid-Liquid Microextraction for Pharmaceutical Analysis	2025	85	Tay, K.S.J.	Malaysia	Crit. Rev. Anal. Chem.	Comprehensive overview of recent DLLME modes utilized in pharmaceutical analysis, detailing their principles, performance, and methodological advancements, including green chemistry considerations and mode comparisons.	[66]

^a^ Note on Coding: Each included systematic review is assigned a unique code (R01–R59) throughout the manuscript. This code serves as the primary identifier for the respective review in all tables and figures to enhance readability and data clarity. ^b^ Indicates book chapters published in edited Elsevier reference books rather than journal articles. ^c^ When the first author had multiple institutional affiliations (e.g., Saudi Arabia–Egypt), only the first listed affiliation was considered for the purpose of geographical and country-level statistical analysis in this umbrella review.

## Data Availability

The data presented in this study are openly available in Open Science Framework (OSF) at 10.1016/J.TRAC.2023.117429.

## Supplementary Materials

The following supporting information can be downloaded at: https://www.mdpi.com/article/10.3390/molecules31111918/s1.
