# Supplementary material for: Twenty Years of Dispersive Liquid–Liquid Microextraction: An Umbrella Review of Methodological Quality, Thematic Evolution, and Roadmap for Evidence Integration in Analytical Chemistry
Source: Molecules. 2026 Jun 2;31(11):1918. doi: 10.3390/molecules31111918 (PMC13257464; doi:10.3390/molecules31111918)
Supplement: Supplementary file 1 [file molecules-31-01918-s001.zip › Supplementary File S1.pdf]

Supplementary File S1

S1.1. Details of the Systematic Search Strategy

Scopus: (TITLE-ABS-KEY("DLLME") OR TITLE-ABS-KEY("Dispersive liquid liquid microextraction") OR TITLE-ABS-KEY("Dispersive liquid-liquid microextraction")) AND (TITLE-ABS-KEY("review") OR TITLE-ABS-KEY("systematic review") OR TITLE-ABS-KEY("meta-analysis") OR TITLE-ABS-KEY("overview") OR TITLE-ABS-KEY("critical review") OR TITLE-ABS-KEY("trends") OR TITLE-ABS-KEY("advances")) AND (LIMIT-TO (PUBYEAR, 2025) OR LIMIT-TO (PUBYEAR, 2024) OR LIMIT-TO (PUBYEAR, 2023) OR LIMIT-TO (PUBYEAR, 2022) OR LIMIT-TO (PUBYEAR, 2021) OR LIMIT-TO (PUBYEAR, 2020) OR LIMIT-TO (PUBYEAR, 2019) OR LIMIT-TO (PUBYEAR, 2018) OR LIMIT-TO (PUBYEAR, 2017) OR LIMIT-TO (PUBYEAR, 2016) OR LIMIT-TO (PUBYEAR, 2015) OR LIMIT-TO (PUBYEAR, 2014) OR LIMIT-TO (PUBYEAR, 2013) OR LIMIT-TO (PUBYEAR, 2012) OR LIMIT-TO (PUBYEAR, 2011) OR LIMIT-TO (PUBYEAR, 2010) OR LIMIT-TO (PUBYEAR, 2009) OR LIMIT-TO (PUBYEAR, 2008) OR LIMIT-TO (PUBYEAR, 2007) OR LIMIT-TO (PUBYEAR, 2006)) AND (LIMIT-TO (DOCTYPE, "re"))

Web of Science (Core Collection): (TS=("DLLME" OR "Dispersive liquid liquid microextraction" OR "Dispersive liquid-liquid microextraction")) AND (TS=("review" OR "systematic review" OR "meta-analysis" OR "overview" OR "critical review" OR "trends" OR "advances")) AND (PY=(2006-2025)) AND (DT=("REVIEW ARTICLE" OR "PROCEEDINGS PAPER" OR "BOOK CHAPTER"))

PubMed: ("Dispersive liquid liquid microextraction"[Title/Abstract]OR ("Dispersive Liquid-Liquid Microextraction" [Title/Abstract] OR "DLLME"[Title/Abstract]) AND ("review" [Publication Type] OR "systematic review" [Publication Type] OR "meta-analysis" [Publication Type] OR "review"[Title/Abstract] OR "overview"[Title/Abstract] OR "critical review"[Title/Abstract] OR "trends" [Title/Abstract] OR "advances" [Title/Abstract]) AND ("2006"[PDat]: "2025"[PDat])

Table S1. Complete Geographical Distribution of Included Systematic Reviews by First Author's Affiliation Country and Continent.

Continent	Country	Article Count	Continent Share (%)	Total Share (%)
Asia	Iran	6	20.7%	10.2%
	China	12	41.4%	20.3%
	Malaysia	2	6.9%	3.4%
	India	1	3.4%	1.7%
	Saudi Arabia	7	24.1%	11.9%
	Japan	1	3.4%	1.7%
	Total	29	100.0%	49.2%
Europe	Spain	11	50.0%	18.6%
	Poland	2	9.1%	3.4%
	Ireland	1	4.5%	1.7%
	Slovakia	5	22.7%	8.5%
	Greece	1	4.5%	1.7%
	Russia	1	4.5%	1.7%
	Italy	1	4.5%	1.7%
	Total	22	100.0%	37.3%
Africa	Egypt	4	100.0%	6.8%
	Total	4	100.0%	6.8%
South America	Brazil	3	100.0%	5.1%
	Total	3	100.0%	5.1%
North America	USA	1	100.00%	1.70%
	Total	1	100.00%	1.70%
**Total**	-	59	-	100.0%

Table S2. The Fifteen most frequently cited core primary studies across included systematic reviews.

Study ID	Inclusion Count	Inclusion Frequency%	Included In Reviews*
10.1016/j.chroma.2006.03.007	25	42.4	R21; R08; R09; R44; R57; R05; R17; R20; R07; R26; R10; R12; R54; R38; R04; R06; R55; R01; R24; R15; R41; R03; R56; R52; R32
10.1016/j.chroma.2008.11.076	13	22.0	R53; R08; R09; R05; R17; R20; R26; R38; R29; R04; R06; R16; R15
10.1016/j.aca.2007.01.007	13	22.0	R05; R17; R20; R26; R22; R02; R06; R37; R50; R01; R18; R15; R03
10.1016/j.chroma.2008.09.111	12	20.3	R53; R08; R05; R17; R20; R26; R12; R29; R06; R55; R15; R03
10.1016/j.jhazmat.2010.09.067	12	20.3	R21; R08; R09; R17; R20; R26; R38; R06; R16; R18; R15; R28
10.1016/j.chroma.2007.04.062	12	20.3	R21; R05; R17; R20; R04; R06; R37; R50; R01; R15; R03; R56
10.1016/j.sab.2009.05.023	12	20.3	R08; R05; R17; R20; R22; R38; R06; R13; R16; R18; R15; R39
10.1016/j.talanta.2009.03.005	12	20.3	R08; R05; R20; R22; R12; R02; R06; R13; R19; R30; R18; R15
10.1016/j.microc.2007.04.003	12	20.3	R09; R05; R20; R22; R10; R02; R06; R13; R37; R18; R15; R03
10.1016/j.chroma.2009.08.012	12	20.3	R09; R05; R17; R20; R26; R38; R35; R04; R06; R16; R15; R52
10.1016/j.ab.2008.05.008	12	20.3	R05; R20; R07; R22; R02; R06; R13; R01; R18; R15; R41; R03
10.1016/j.chroma.2007.05.006	11	18.6	R14; R21; R09; R05; R17; R20; R04; R06; R01; R15; R03
10.1002/jssc.200900109	11	18.6	R21; R17; R20; R26; R12; R38; R33; R04; R06; R15; R27
10.1016/j.chroma.2007.09.002	11	18.6	R21; R05; R17; R20; R04; R06; R36; R50; R01; R15; R03
10.1002/jssc.201000088	11	18.6	R21; R08; R17; R20; R29; R06; R25; R36; R50; R15; R32

*The codes used for the axes (R01–R59) correspond to the unique identifiers assigned to each systematic review, as detailed in Table 1.
