# Supplementary material for: Twenty Years of Dispersive Liquid–Liquid Microextraction: An Umbrella Review of Methodological Quality, Thematic Evolution, and Roadmap for Evidence Integration in Analytical Chemistry
Source: Molecules. 2026 Jun 2;31(11):1918. doi: 10.3390/molecules31111918 (PMC13257464; doi:10.3390/molecules31111918)
Supplement: Supplementary file 1 [file molecules-31-01918-s001.zip › Suppl. FileS5_2_Final-Overlap-Analysis-Tool.html]

Systematic Review Overlap Analysis Tool


# Systematic Review Overlap Analysis Tool

Export Excel

Export Word

Save Data

Select Systematic Reviews CSV File:


Start Overlap Analysis

### Required CSV Format:

Code,DOI,Title,DOIs/PMIDs/WOS IDs of Included Primary Studies  
R01,10.1234/rev1,"Review Title 1","study1;study2;study3;study4"  
R02,10.1234/rev2,"Review Title 2","study2;study4;study5;study6"  
R03,10.1234/rev3,"Review Title 3","study1;study3;study6;study7"

Analyzing Systematic Reviews Overlap...

Calculating C-Index, Overlap Matrix, and Setup Diagrams

--

Corrected Covered Area

CCA Index

--

Unique Studies

Primary Research

--

Total Inclusions

Study References

--

Systematic Reviews

Analyzed

--

Core Studies

≥2 Reviews

--

Mean Overlap

Percentage


### Pairwise C-Index Matrix of Primary Studies

**C-Index Interpretation:** Values range from 0 (no overlap) to 1 (complete overlap). Shows pairwise overlap between systematic reviews.

### Overlap Matrix (Count of Shared Primary Studies)

### Core Primary Studies Analysis

| Primary Study ID | Inclusion Count | Inclusion Frequency | Included In Systematic Reviews |
| --- | --- | --- | --- |

AQAIMPA Universidad de La Laguna

Systematic Review Research Group

Systematic Review Overlap Analysis Tool

Developed for Academic Research Purposes

University Website

Contact
