# Supplementary material for: Twenty Years of Dispersive Liquid–Liquid Microextraction: An Umbrella Review of Methodological Quality, Thematic Evolution, and Roadmap for Evidence Integration in Analytical Chemistry
Source: Molecules. 2026 Jun 2;31(11):1918. doi: 10.3390/molecules31111918 (PMC13257464; doi:10.3390/molecules31111918)
Supplement: Supplementary file 1 [file molecules-31-01918-s001.zip › Supplementary File S3_Adapted AMSTAR 2.pdf]

Universidad de La Laguna

AQAIMPA

Supplementary Materials, File S3.

S3.1. Adapted AMSTAR 2- A Critical Appraisal Tool for Systematic Reviews in Dispersive Liquid–Liquid Microextraction (DLLME)

Introduction

The AMSTAR 2 tool (A MeaSurement Tool to Assess Systematic Reviews) was originally designed to critically appraise the methodological quality of systematic reviews, particularly those involving randomized controlled trials (RCTs) or non-randomized studies of interventions (NRSI), or both. Unlike tools that generate a single "overall score," AMSTAR 2 focuses on identifying *critical weaknesses in specific domains* that could substantially reduce confidence in the results of a systematic review.

In analytical chemistry — especially in methods such as *Dispersive Liquid–Liquid Microextraction (DLLME)* — most reviews are of a validatory and comparative nature, while fully structured professional systematic reviews are relatively rare.

This adapted checklist aligns AMSTAR 2 with the specific characteristics of DLLME research, including:

- the types of data typically reported,
- the sources of potential bias unique to analytical method development, and
- the synthesis approaches used to summarize findings.

The aim is to give analytical chemistry researchers a practical, methodologically sound tool to assess the quality and reliability of DLLME-related reviews.

Adapted AMSTAR 2 Checklist for DLLME Review Articles

1. Did the research questions and inclusion criteria for the review contain the PICO components?

(PICO: Population, Intervention, Comparator, Outcome)

- **Purpose of the item:**To ensure the review has a clear, well-defined research question that allows for the assessment of study selection, the feasibility of combining results, and the applicability of findings.
- **Adaptation for DLLME:**In DLLME research, PICO components are interpreted as follows:
  - ✓ **(P) Population / Sample Matrix:** The type of sample matrix under investigation (e.g., water, soil, biological fluids, food samples, etc.).
  - ✓ **(I) Intervention / Method:** The DLLME procedure used, including key parameters such as type and volume of extraction solvent, type and volume of disperser solvent, extraction time, centrifugation speed and time, stirring speed and time, temperature, pH, ionic strength.
  - ✓ **(C) Comparator / Alternative Methods or No Intervention:** Comparison with other established extraction methods, validated modified DLLME procedures, samples spiked with known amounts of target analyte, or control samples without the target analyte.
  - ✓ **(O) Outcome / Target Analyte(s) and Performance Criteria:** Quantification of the target analyte(s) and method performance metrics (e.g., extraction efficiency, enrichment factor, and figures of merit under optimal conditions — including sensitivity measured by limit of detection (LOD) and limit of quantification (LOQ), linearity assessed by the mathematical model relating concentration to analytical signal, accuracy evaluated by recovery, and precision assessed by repeatability and reproducibility).
- **Scoring guidance:**
  - ✓ **Yes:** All four PICO components (Population/Matrix, Intervention/DLLME Method, Comparator, Outcome) are clearly described somewhere in the review report (e.g., in the abstract, introduction, or methods section).
  - ✓ **Partial Yes:** At least two components, (P) Population/Matrix and (I) Intervention/Method, are clearly described somewhere in the review.
  - ✓ **No:** The PICO components are not clearly defined.

2. Did the review report explicitly state that the review methods were established prior to conducting the review, and did it justify any significant deviations from the protocol?

(Critical domain)

- **Purpose of the item:** To ensure that the systematic review was conducted according to a pre-defined, written, and (ideally) registered protocol, thereby reducing the risk of bias during the review process.
- **Adaptation for DLLME:** While formal protocol registration in PROSPERO (primarily for clinical reviews) or OSF is less common in chemical sciences, authors should clearly state that they worked from a written protocol including:
  - ✓ review questions,
  - ✓ search strategy,
  - ✓ inclusion/exclusion criteria, and
  - ✓ risk of bias assessment.

  Any deviations from this protocol must be justified.
- **Scoring guidance:**
  - ✓ **Yes:** Authors state that they had a written protocol including review questions, search strategy, inclusion/exclusion criteria, risk of bias assessment, meta-analysis/synthesis plan (if applicable), and a plan to explore heterogeneity, *and* that the protocol was registered, with all deviations justified.
  - ✓ **Partial Yes:** Authors state that they had a written protocol including review questions, search strategy, inclusion/exclusion criteria, and risk of bias assessment.
  - ✓ **No:** No explicit statement about a pre-defined protocol.

---

3. Did the review authors explain their selection of the study designs for inclusion in the review?

- **Purpose of the item:** To ensure clarity on the specific types of primary studies included in the review and the rationale behind their inclusion. For example, only primary studies using a certain extraction solvent (e.g., DES, ionic liquids) in DLLME, studies quantifying specific analytes, studies using DLLME in specific matrices, or those comparing DLLME with other extraction techniques.
- **Adaptation for DLLME:** Authors should explain why they included only:
  - ✓ studies quantifying specific analytes or matrices using DLLME,
  - ✓ studies using particular extraction or disperser solvents,
  - ✓ DLLME optimization studies,
  - ✓ DLLME hardware development studies,
  - ✓ coupling DLLME with other analytical instrumentation, or
  - ✓ specific comparative studies.

  For example, a review may focus solely on DLLME with bio-friendly solvents or solvent-removal techniques.
- **Scoring guidance:**
  - ✓ **Yes:** A clear rationale for including/excluding certain types of studies (e.g., only method development, only applications to certain matrices, or both).
  - ✓ **No:** No rationale for selection of study designs.

---

4. Did the review authors use a comprehensive literature search strategy?

(Critical domain)

- **Purpose of the item:** To ensure that all relevant studies addressing the review question were identified, thereby reducing selection and publication bias.
- **Adaptation for DLLME:**
  - ✓ At least two bibliographic databases relevant to analytical chemistry (e.g., ScienceDirect, Web of Science, Scopus, Google Scholar, PubMed) should be searched.

- ✓ Full search keywords and/or strategy should be reported.
- ✓ Searches should be supplemented by checking reference lists of identified studies and consulting experts in DLLME.
- ✓ Grey literature (e.g., theses, technical reports, patents) should be searched where relevant, especially for emerging or niche DLLME applications.
- ✓ Language restrictions should be justified.
- ✓ Searches should have been conducted within 24 months prior to review completion.

- **Scoring guidance:**
  - ✓ **Yes:** All "Partial Yes" criteria plus: reference list checking, registry searching, expert consultation, grey literature search, and search within the last 24 months.
  - ✓ **Partial Yes:** At least two databases searched, search terms/strategy reported, and publication restrictions justified.
  - ✓ **No:** "Partial Yes" criteria not met.

---

5. Did the review authors perform study selection in duplicate?

- **Purpose of the item:** To reduce the risk of bias in the study selection stage by using two independent reviewers.
- **Adaptation for DLLME:** At least two reviewers should independently screen titles, abstracts, and full texts for DLLME study eligibility. A consensus process should resolve disagreements.
- **Scoring guidance:**
  - ✓ **Yes:** At least two reviewers independently agreed on eligible studies and reached consensus on inclusion; or two reviewers screened a sample of studies achieving ≥80% agreement, with the remainder screened by one reviewer.
  - ✓ **No:** Study selection not performed in duplicate.

---

6. Did the review authors perform data extraction in duplicate?

- **Purpose of the item:** To reduce the risk of bias and data errors during extraction from included studies.
- **Adaptation for DLLME:** At least two independent reviewers should extract key data from each study (e.g., extraction efficiency, enrichment factor, LOD/LOQ, linear range, optimal DLLME conditions, matrix details, analyte type). A consensus process should resolve disagreements.
- **Scoring guidance:**
  - ✓ **Yes:** At least two reviewers reached consensus on extracted data; or two reviewers extracted data from a sample of studies with ≥80% agreement, with the remainder extracted by one reviewer.
  - ✓ **No:** Data extraction not performed in duplicate.

---

7. Did the review authors provide a list of excluded studies and justify the exclusions?

(Critical domain)

- **Purpose of the item:** To increase transparency and detect potential selection bias.
- **Adaptation for DLLME:** A full list of potentially relevant studies read in full text but excluded from the review should be provided, with clear reasons for exclusion (e.g., not focused on DLLME, irrelevant matrix, unsuitable analyte, insufficient methodological detail, lacking primary data).
- **Scoring guidance:**
  - ✓ **Yes:** List of all excluded potentially relevant studies provided, with justification for each.
  - ✓ **Partial Yes:** List provided but without justifications.
  - ✓ **No:** No list of excluded studies provided.

---

8. Did the review authors describe the included studies in adequate detail?

- **Purpose of the item:** To provide sufficient information for the reader to assess the appropriateness of the studies and the applicability of results.
- **Adaptation for DLLME:** Descriptions should include:
  - ✓ analytes,
  - ✓ sample matrices,
  - ✓ DLLME method parameters (solvents, volumes, temperature, pH, ionic strength, instrumentation),
  - ✓ study design (optimization, validation, application), and
  - ✓ reported performance metrics (LOD, LOQ, extraction efficiency, enrichment factor, recovery, precision, accuracy).

  Also, study setting and follow-up duration (if relevant) should be mentioned.
- **Scoring guidance:**
  - ✓ **Yes:** All "Partial Yes" criteria (population/matrix, interventions/methods, comparators, outcomes, study designs) plus additional details (matrix specifics, method parameters, comparator details, setting, follow-up).
  - ✓ **Partial Yes:** Description of population/matrix, interventions/methods, comparators, outcomes, and study designs.
  - ✓ **No:** Insufficient detail provided.

---

9. Did the review authors use a satisfactory technique for assessing the risk of bias (RoB) in individual studies that were included in the review?

(Critical domain)

- **Purpose of the item:** To identify methodological flaws in primary studies that could bias their results.
- **Adaptation for DLLME:** In analytical method development, "bias" differs from clinical contexts. Reviews should assess potential bias sources specific to DLLME, including:

1. Failure to use Design of Experiments (DOE) instead of one-variable-at-a-time (OVAT) approaches in optimization.
2. Inadequate method validation, such as absence of statistical tests for figures of merit (accuracy, precision, sensitivity — linearity, LOD, LOQ — and recovery).
3. Validation not based on recognized, reputable protocols.
4. Reliance on visual inspection of plots without statistical inference.
5. Lack of suitable reference materials or external validation.
6. Not addressing interferences commonly encountered in DLLME applications.
7. Omission of key experimental parameters affecting performance (e.g., laboratory humidity, environmental temperature control).

- **Scoring guidance:**
  - ✓ **Yes:** Authors addressed items 1–7 explicitly, and also evaluated confounding factors, selection bias, methods for determining exposures and outcomes, and selective outcome reporting from multiple measurements/analyses.
  - ✓ **Partial Yes:** Authors explicitly addressed at least items 1, 2, and 3; items 4–7 were either omitted or weakly reported.
  - ✓ **No:** "Partial Yes" mandatory items not met; bias not satisfactorily assessed.

---

10. Did the review authors report on the sources of funding for the studies included in the review?

- **Purpose of the item:** To identify potential conflicts of interest in primary studies that could affect reported results.
- **Adaptation for DLLME:** Review authors should document the funding sources of each included study or note when such information is absent in study reports, as commercial sponsorship may influence reported method performance.
- **Scoring guidance:**
  - ✓ **Yes:** Funding sources for all included primary studies reported (or explicitly stated as not provided by study authors).

- ✓ **No:** Funding sources not reported.

---

11. If a meta-analysis was justified, did the review authors use appropriate methods for statistical combination of results?

(Critical domain)

- **Purpose of the item:** To evaluate the appropriateness of data combination methods and handling of heterogeneity.
- **Adaptation for DLLME:** Most DLLME reviews are narrative/qualitative with no statistical pooling. If no meta-analysis was done: *Not applicable*.

If meta-analysis was performed, authors should:

- ✓ justify its use (e.g., pooling extraction efficiency or LODs),
- ✓ apply suitable weighting techniques and adjust for heterogeneity,
- ✓ adjust pooled estimates from non-randomized studies for confounding factors (e.g., matrix type, interferences), and
- ✓ report separate summaries if both RCTs and non-RCTs (rare in DLLME) are included.

- **Scoring guidance:**
  - ✓ **Yes:** Above criteria met for the appropriate study type (NRSI).
  - ✓ **No:** Above criteria not met.
  - ✓ **Not applicable:** No quantitative synthesis conducted.

---

12. If a meta-analysis was performed, did the review authors assess the potential impact of RoB in individual studies on the results of the meta-analysis or other evidence syntheses?

- **Purpose of the item:** To determine whether the review accounted for the influence of identified biases on the overall findings.
- **Adaptation for DLLME:** If no meta-analysis: *Not applicable*.

If meta-analysis performed: authors should analyze the impact of RoB on summary effect estimates (e.g., sensitivity analysis excluding high-RoB studies, or assessing impact of DLLME-specific biases like OVAT vs DOE).

- **Scoring guidance:**
  - ✓ **Yes:** If pooled estimates included studies with varying RoB, authors conducted analyses to assess RoB's effect on summary estimates.
  - ✓ **No:** Meta-analysis conducted but RoB impact not assessed.
  - ✓ **Not applicable:** No quantitative synthesis conducted.

---

13. Did the review authors account for RoB in individual studies when interpreting/discussing the results of the review?

(Critical domain)

- **Purpose of the item:** To ensure interpretation of review results considers limitations caused by biases in primary studies.
- **Adaptation for DLLME:** Even in narrative reviews, authors should discuss the influence of biases identified in Item 9 — such as lack of DOE, absence of statistical validation, inadequate protocol-based validation, reliance on visual assessment — on the credibility of figures of merit and on determining optimal independent variables in DLLME.
- **Scoring guidance:**
  - ✓ **Yes:** Discussion/conclusions explicitly mention methodological weaknesses (reinterpreted as "risk of bias") in primary studies and their effect on reliability of findings.
  - ✓ **No:** Insufficient discussion of RoB's effect on review results.

---

14. Did the review authors provide a satisfactory explanation for, and discussion of, any heterogeneity observed in the results of the review?

- **Purpose of the item:** To identify and explain sources of variability in study results for more accurate interpretation.
- **Adaptation for DLLME:** Heterogeneity is common in DLLME (e.g., in LODs, enrichment factors, extraction efficiencies). Authors should explore causes such as:
  - ✓ different calculation methods,
  - ✓ matrix type and amount,
  - ✓ analyte properties,
  - ✓ method parameters (solvent type/volume, temperature, pH, ionic strength),
  - ✓ instrumentation used,
  - ✓ different validation protocols, and
  - ✓ type of bias present (Item 9).
- **Scoring guidance:**
  - ✓ **Yes:** No significant heterogeneity observed; or heterogeneity explored and its impact discussed.
  - ✓ **No:** Heterogeneity not examined or explained.

---

15. If they performed a quantitative synthesis, did the review authors carry out an adequate investigation of publication bias (small-study bias) and discuss its likely impact on the results of the review?

(Critical domain)

- **Purpose of the item:** To determine whether the review attempted to detect publication bias, where "positive" studies are more likely to be published.
- **Adaptation for DLLME:** As with Item 11, quantitative syntheses are rare in DLLME reviews. If no quantitative synthesis: *Not applicable*.

If performed: authors should use statistical tests or graphical methods (e.g., funnel plot if ≥10 studies) to detect publication bias, and discuss its possible effects (e.g., smaller studies with poorer performance — higher LOD, lower efficiency — being underrepresented).

- **Scoring guidance:**
  - ✓ **Yes:** Publication bias tested and its possible effects discussed.
  - ✓ **No:** Publication bias not assessed or discussed.
  - ✓ **Not applicable:** No quantitative synthesis conducted.

---

16. Did the review authors report any potential sources of conflict of interest, including any funding they received for conducting the review?

- **Purpose of the item:** To identify potential conflicts of interest of review authors that might influence planning, conduct, or reporting of the review.
- **Adaptation for DLLME:** Authors should report funding sources for the review itself and disclose any other relevant relationships (e.g., membership in corporate advisory boards, travel grants, shareholding in companies producing analytical equipment/chemicals, extensive prior publications in the same niche DLLME field).
- **Scoring guidance:**
  - ✓ **Yes:** Authors declare no competing interests or describe funding and how potential conflicts were managed.
  - ✓ **No:** No disclosure of potential conflicts of interest.

Important Notes for Analytical Chemistry Users

- **Emphasis on Qualitative Approach:** As established in preliminary assessments, most reviews in *Dispersive Liquid–Liquid Microextraction (DLLME)* are qualitative narratives. Consequently, items related to meta-analysis (Items 11, 12, and 15) are often *"Not applicable"*.
  However — even in purely qualitative reviews — thorough risk of bias (RoB) assessment (Item 9) and explicit consideration of RoB in interpreting results (Item 13) are *critical domains* that must not be overlooked.
- **Adapting Bias Assessment:** Understanding how biases in analytical research (e.g., failure to use DOE, inadequate validation, reliance on visual inspection) correspond to

the broader RoB concepts in AMSTAR 2 is essential for an accurate, field-relevant evaluation. This mapping helps prevent underestimating methodological flaws that could undermine reported figures of merit.

- **Transparency in Reporting:** Regardless of whether the review is quantitative or qualitative, transparency in reporting methodological parameters and decision-making throughout the review process is crucial.

This includes:

- ✓ clearly documenting search strategies,
- ✓ justifying inclusion/exclusion decisions,
- ✓ providing complete methodological details of included studies, and
- ✓ explaining any methodological compromises or deviations from the planned protocol.

- **Practical Implication:** This adapted checklist is intended to help analytical chemistry researchers and practitioners evaluate DLLME reviews with both methodological rigor and domain-specific insight. Applying it consistently will improve the reliability and reproducibility of conclusions drawn from such reviews — especially when guiding method development, optimization, and application in complex matrices.

S3.2. Methodology for Assessing Overall Confidence Rating (AMSTAR 2)

AMSTAR 2 is **not** designed to generate a single overall numerical "score" for systematic reviews. Instead, it employs a qualitative **"Overall Confidence Rating"** based on the identification of weaknesses, particularly within specific **"Critical Domains"**. This approach emphasizes that critical flaws can significantly diminish the confidence in a systematic review, regardless of its performance on other items.

The procedure for determining the Overall Confidence Rating is as follows:

1. Assessment of Individual Items

- Each of the 16 items on the AMSTAR 2 checklist is evaluated and assigned a response of **"Yes"**, **"Partial Yes"**, or **"No"**.
- **"Yes"** indicates complete adherence to the standard.
- **"Partial Yes"** indicates partial adherence for certain items, which is considered valuable to identify.
- **"No"** is assigned if the information is not provided in the review, meaning the authors do not receive the benefit of the doubt.

2. Identification of Critical Domains

AMSTAR 2 identifies seven specific items as **"Critical Domains"** that can profoundly impact the validity of a review and its results. These domains include:

- **Item 2:** Registration of a protocol prior to the commencement of the review.
- **Item 4:** Adequacy of the literature search strategy.
- **Item 7:** Justification for excluding individual studies.
- **Item 9:** Assessment of the Risk of Bias (RoB) in individual studies included in the review.
- **Item 11:** Appropriateness of meta-analysis methods (if a meta-analysis was performed).
- **Item 13:** Consideration of the risk of bias when interpreting the review results.
- **Item 15:** Assessment of the presence and likely impact of publication bias (if quantitative synthesis was performed).

Note on Application:

While these are the suggested critical items, assessors may choose to add or substitute other critical domains, or consider some of the listed critical items less important based on the specific context of the review. For example, if a review is limited to only high-quality Randomized Controlled Trials (RCTs), RoB-related items might be less critical. Furthermore, Items 11 and 15 are not applicable if a meta-analysis was not performed. The appraisal team must establish consensus on the application of the AMSTAR 2 items, including the relevant PICO components and potential sources of bias.

3. Assignment of the Overall Confidence Rating

The overall confidence in the results of the systematic review is rated using a four-level scheme, primarily based on the presence and number of weaknesses in the critical domains:

Overall Confidence Rating	Criteria	Implication
**High Confidence**	The review has no non-critical weaknesses or only one non-critical weakness.	Indicates that the systematic review provides an accurate and comprehensive summary of the results of available studies addressing the question of interest.
**Moderate Confidence**	The review has more than one non-critical weakness but no critical flaws.	The review may provide an accurate summary of the results of the included studies. Note: Multiple non-critical weaknesses can reduce confidence and may warrant downgrading to "Low Confidence".
**Low Confidence**	The review has one critical flaw (with or without additional non-critical weaknesses).	Indicates that the review may not provide an accurate and/or comprehensive summary of available studies addressing the question of interest.
**Critically Low Confidence**	The review has more than one critical flaw (with or without non-critical weaknesses).	This review should not be relied upon to provide an accurate and comprehensive summary of available studies.

4. Practical Implementation Steps

In practice, the steps for assigning the rating are as follows:

1. Evaluate each of the 16 items and assign a "Yes," "Partial Yes," or "No" response.
2. Identify which items are considered **"Critical"** for the specific review being appraised. (Adapt the default list of critical domains if necessary).
3. Determine if any of the critical items received a **"No"** or **"Partial Yes"** response (indicating a weakness or flaw).
4. Count the number of **critical flaws**.
5. Count the number of **non-critical weaknesses**.
6. Apply the rules outlined in the table above (Section 3) to determine the final Overall Confidence Rating.

It is essential that assessors do **not** combine the rating of individual items to create an overall numerical score, but rather consider the potential impact of each inadequate rating. If further information (e.g., from the review authors) could resolve an initial "No" rating, it may provide reassurance and alter the assessment.
